# A Novel Comb Architecture for Enhancing the Sensitivity of Bulk Mode Gyroscopes

**DOI:** 10.3390/s131216641

**Published:** 2013-12-04

**Authors:** Mohannad Y. Elsayed, Frederic Nabki, Mourad N. El-Gamal

**Affiliations:** 1 Department of Electrical and Computer Engineering, McGill University, Montréal, Québec H3A 0E9, Canada; E-Mail: mohannad.elsayed@mail.mcgill.ca; 2 Department of Computer Science, Université du Québec à Montréal, Montréal, Québec H3C 3P8, Canada; E-Mail: nabki.frederic@uqam.ca

**Keywords:** MEMS, resonant gyroscopes, bulk-mode, sensitivity enhancement

## Abstract

This work introduces a novel architecture for increasing the sensitivity of bulk mode gyroscopes. It is based on adding parallel plate comb drives to the points of maximum vibration amplitude, and tuning the stiffness of the combs. This increases the drive strength and results in a significant sensitivity improvement. The architecture is targeted for technologies with ∼100 nm transducer gaps in order to achieve very high performance devices. In this work, this sensitivity enhancement concept was implemented in SOIMUMPs, a commercial relatively large gap technology. Prototypes were measured to operate at frequencies of ∼1.5 MHz, with quality factors of ∼33,000, at a 10 mTorr vacuum level. Measurements using discrete electronics show a rate sensitivity of 0.31 μV/°/s, corresponding to a capacitance sensitivity of 0.43 aF/°/s/electrode, two orders of magnitude higher than a similar design without combs, fabricated in the same technology.

## 1. Introduction

Micro-machined gyroscopes are receiving more and more attention in the sensing community. This is due to their small size, which enable their use in numerous consumer electronics applications (e.g., image stabilization in digital cameras, motion sensing, *etc.*). Gyros are also used in navigation systems for robotic, military, aeronautic and space applications, providing a significant opportunity for the growth of their micro-machined implementations.

As highlighted in [1], bulk mode gyroscopes are capable of achieving superior performance compared to flexural mode ones. These gyros operate in higher order resonant modes, and thus their operating frequencies are generally in the MHz range—three orders of magnitude higher than flexural mode gyros. Notably, higher order modes experience less thermoelastic damping than flexural modes. Moreover, they can achieve very high quality factors (∼50,000) even in atmospheric pressure, as air damping has little impact on their operation due to the high stiffness of their structures. Also, bulk mode gyroscopes exhibit orders of magnitude higher bandwidths than flexural mode ones, thus relaxing the need for drive-sense mode matching and expensive vacuum packaging, which are both mandatory for the operation of flexural mode gyroscopes.

In addition, bulk mode gyroscopes generally exhibit lower mechanical noise than flexural ones. The mechanical noise of a vibratory gyroscope is given by:

(1) $$\Omega_{z(\text{Brownian})}\propto \frac{1}{q_{\text{drive}}}\sqrt{\frac{4k_{B}T}{\omega_{0}MQ_{\text{effect}-\text{sense}}}}$$

where *q_drive_* is the drive mode vibration amplitude, *k_B_* is the Boltzmann constant, *T* is the absolute temperature, ω_0_ is the resonance frequency, *M* is the mass, and *Q_effect-sense_* is the effective quality factor [1]. Today, state-of-the-art vibratory gyroscopes operating at lower frequencies (<100 kHz) rely on increasing the proof mass or the vibration amplitude to improve their noise performance. They require low vacuum operation (<10 mTorr) to achieve high quality factors (<10,000) that are ultimately limited by thermoelastic damping. Enhancing the noise performance of gyros without the need for increasing the mass or vibration amplitude is of great interest, as these parameters are limited mainly by design area and power concerns. From Equation (1), it is clear that raising the resonance frequency and the quality factor, as allowed by bulk mode architectures, improves the noise performance of a gyroscope significantly.

Different bulk mode gyroscopes to take advantage of these improved characteristics have recently been published. In [1,2], a circular disk architecture was introduced in (100) and (111) silicon, and a circular disk gyro with spokes was suggested in [3]—it combined flexural and bulk modes, and achieved a large dynamic range. Bulk mode gyros (e.g., [1–3]) and resonators (e.g., [4–7]) typically utilize very small transducer gaps (e.g., 200 nm) between the center resonating element and the electrodes. In [8], a 3 μm gap dodecagon bulk mode gyroscope design utilizing the same fabrication technology as here, but without comb drives, was reported. This device's sensitivity was significantly less than the previously mentioned devices due to its relatively large transducer gap, mainly dictated and limited by the technology used.

This work introduces a novel architecture for raising the sensitivity of bulk mode gyroscopes. It is based on adding parallel plate comb drives to the points of maximum vibration amplitude, and tuning the stiffness of these combs. This increases the drive strength and results in significant improvement in the sensitivity. The architecture is well-suited for technologies with ∼100 nm transducer gaps in order to achieve very high performance devices. In this work, as a proof of concept, the idea was also implemented in a commercial relatively large gap technology (SOIMUMPs) in order to outline the sensitivity improvement possible through the proposed method in a widely available standard bulk micromachining technology. The design is composed of a central dodecagon disk structure with added parallel plate comb drives. Adding combs connected to the central disk structure increases the drive strength and results in two orders of magnitude higher sensitivity than the design presented in [8]. This enables the fabrication of potentially high performance bulk-mode gyroscopes in standard commercial MEMS technologies. Also, the gyro here is amenable for fabrication in select above-IC technologies, e.g., [9–11]. The concept was introduced briefly in [12]. Full details about the design, fabrication, and testing are presented in this work.

The paper is structured such that the operating principle of the device and its design are first described. Simulation results are then reported, and are followed by measurement results of the fabricated device. The device performance is then discussed, and subsequently a conclusion is presented.

## 2. Operating Principle

Generally, vibratory gyroscopes rely in their operation on the Coriolis effect, where if a body of mass (*m*) is moving with a velocity (*υ*) in a certain direction and is acted upon by a rotation rate (Ω) around another direction, a force is generated that will move the body in a direction perpendicular to both the direction of the original motion and the axis of rotation. This Coriolis force, *F_c_*, is given by Equation (2) [13]:

(2) $$F_{c}=2m(\upsilon \times \omega )$$

**Figure 1.**
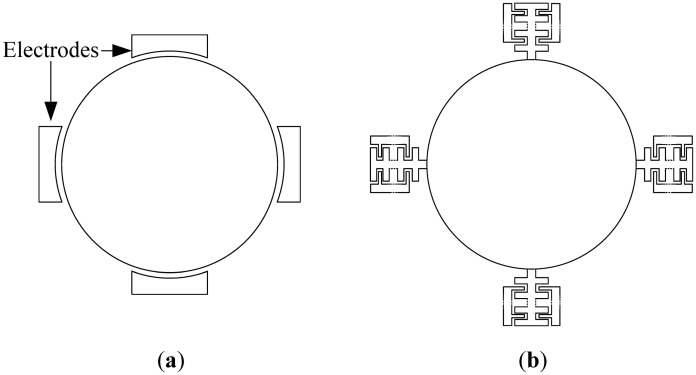
Simplified illustration for the (**a**) typical disk gyroscope, and (**b**) the proposed combined disk-comb gyroscope (electrodes are positioned symbolically around the disk structure).

Bulk mode disk devices are composed of a main central disk structure (circular or polygonal), which vibrates in a higher order bulk mode. Outer electrodes are used for electrostatic actuation of the structure in the drive mode. When the device is subject to rotation, the Coriolis force excites a different mode than the drive mode, the sense mode. The resulting vibration is then detected through the sense electrodes, enabling the gyroscope operation. Traditionally, to drive and sense the disk vibration, electrodes are directly placed at vibrational areas around the disk, as shown in Figure 1a.

**Figure 2.**
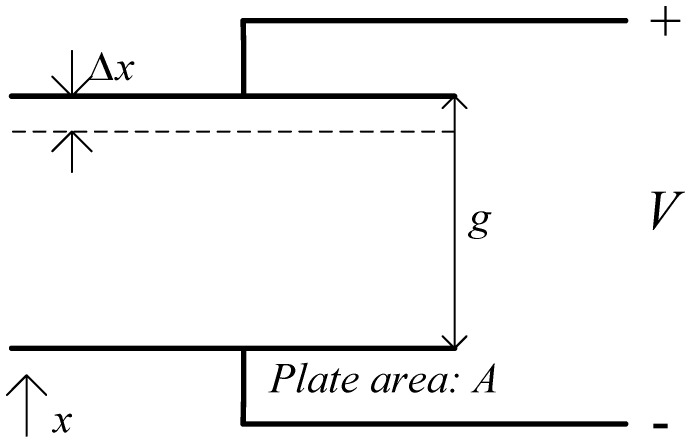
Simplified illustration for a parallel plate capacitor.

The idea proposed here extends the central resonating disk structure by adding parallel plate comb drives with a variable gap configuration to the parts of the disk which exhibit the maximum vibration amplitude in the drive and sensor resonant modes, as shown in simplified form in Figure 1b. This increases the drive strength and enhances the device sensitivity. This sensitivity improvement is due to the increased electrostatic force and capacitance change that result because of the larger electrode surface area. Equations (3) and (4) outline the impact of electrode area on the force and capacitance, in relation to a parallel place capacitor shown in Figure 2 [14]:

(3) $$F_{\text{electrostatic}}=\frac{1}{2}\frac{dc}{dx}V^{2}=\frac{1}{2}\frac{{ɛ}_{0}A}{g^{2}}V^{2}$$

(4) $$\begin{array}{cc} \Delta C\approx \frac{{ɛ}_{0}A}{g^{2}}\Delta x, & \Delta x\ll g \end{array}$$

**Figure 3.**
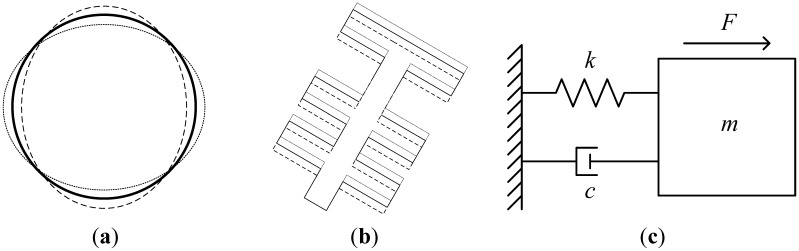
(**a**) Resonating disk structure, (**b**) resonating comb structure, and (**c**) resonant mass-spring-damper model.

**Figure 4.**
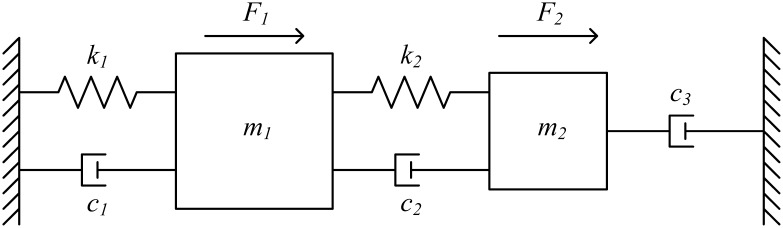
Double mass-spring-damper system model for the combined disk-comb structure.

Generally, any resonating structure, including the disk resonator and the comb resonating structure, can be modeled with a mass-spring-damper system as shown in Figure 3. In order to model the combined resonating system, the mass-spring-damper models for both the disk and the comb resonators are combined to give the double mass-spring-damper system shown in Figure 4, which will be used to illustrate the operating principle of the combined disk-comb device. *k_1_* and *k_2_* are the spring constants of the disk (primary structure) and the comb (secondary structure), respectively; *m_1_* and *m_2_* are the masses of the disk and the comb, respectively; and *c_1_*, *c_2_*, and *c_3_* are the different damping coefficients acting on the system. *F_1_* is the electrostatic force acting on the disk faces directly, and *F_2_* is the total electrostatic force of each comb drive. *x_1_* and *x_2_* are the displacements of the primary and secondary masses, respectively.

The system can be described as follows:

(5) $$F_{1}-k_{1}x_{1}(t)-c_{1}{\dot{x}}_{1}(t)-k_{2}\left(x_{1}(t)-x_{2}(t)\right)-c_{2}\left({\dot{x}}_{1}(t)-{\dot{x}}_{2}(t)\right)=m_{1}{\ddot{x}}_{1}(t)$$

(6) $$F_{2}-k_{2}\left(x_{2}(t)-x_{1}(t)\right)-c_{2}\left({\dot{x}}_{2}(t)-{\dot{x}}_{1}(t)\right)-c_{3}{\dot{x}}_{2}(t)=m_{2}{\ddot{x}}_{2}(t)$$

These equations can be simplified giving:

(7) $$F_{1}-(k_{1}+k_{2})x_{1}(t)+k_{2}x_{2}(t)-\left(c_{1}+c_{2}\right){\dot{x}}_{1}(t)+c_{2}{\dot{x}}_{2}(t)=m_{1}{\ddot{x}}_{1}(t)$$

(8) $$F_{2}+k_{2}x_{1}(t)-k_{2}x_{2}(t)+c_{2}{\dot{x}}_{1}(t)-\left(c_{2}+c_{3}\right){\dot{x}}_{2}(t)=m_{2}{\ddot{x}}_{2}(t)$$

Since the force of a given comb finger is, by design, equivalent to the force applied onto a disk face, the total comb force is applied to the disk face, the following relationship between *F_1_* and *F_2_* can be written:

(9) $$F_{2}=nF_{1}$$

where n is the number of fingers in each comb drive. By solving Equations (7) and (8) simultaneously and taking into consideration Equation (9), the system transfer function can be reached as given by Equation (10):

(10) $${\frac{X_{1}(S)}{F_{1}(S)}|}_{\text{double}}=\frac{m_{2}S^{2}+\left((n+1)c_{2}+c_{3}\right)S+(n+1)k_{2}}{\left[m_{1}m_{2}S^{4}+(m_{1}c_{2}+m_{1}c_{3}+m_{2}c_{1}+m_{2}c_{2})S^{3}+(m_{1}k_{2}+m_{2}k_{1}+m_{2}k_{2}+c_{1}c_{2}+c_{1}c_{3}+c_{2}c_{3})S^{2}+(c_{1}k_{2}+c_{2}k_{1}+c_{3}k_{1}+c_{3}k_{2})S+k_{1}k_{2}\right]}$$

**Figure 5.**
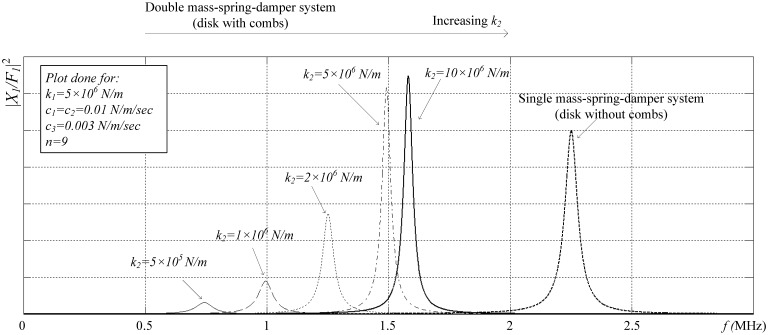
|X_1_/F_1_|^2^ for the disk-comb combined structure (double spring-mass-damper system for different secondary spring constants), and for the disk structure (single spring-mass-damper system).

Figure 5 shows the magnitude of this transfer function versus frequency for different values of *k_2_*, with all other parameters kept constant, and for the mode utilized in our design, where the two masses exhibit in-phase motion. The transfer function of a single mass-spring-damper system (*m_1_*-*k_1_*-*c_1_*) is also plotted for comparison and is given by Equation (11):

(11) $${\frac{X_{1}(S)}{F_{1}(S)}|}_{\sin\mathrm{gle}}=\frac{n+1}{m_{1}S^{2}+c_{1}S+k_{1}}$$

The equivalent force of the comb *(n* + *1)F_1_* is applied to the single spring-mass-damper system for the comparison. From Figure 5, it is clear that when the secondary spring constant is small compared to the primary spring constant, the primary mass displacement at resonance is small, as the force on the secondary mass is not conveyed effectively to the primary mass. As the secondary spring constant is increased, the force is coupled more effectively to the disk and the displacement increases even over the displacement in a single spring-mass-damper system when the same total force is applied directly to the primary mass. Accordingly, the disk drive and sense coupling is increased in this work by carefully designing the spring constant of the comb drive structure so that its driving force (and sensing) enhances optimally the performance of the bulk resonant structure. It is well-suited to bulk mode devices built in sub-micron gap technologies (∼100 nm), to produce very high performance devices. In this work, the idea is implemented in a commercial 3 μm gap technology (SOIMUMPs), and the results are compared to other designs in the literature, as a proof of concept for our novel architecture.

## 3. Design

**Figure 6.**
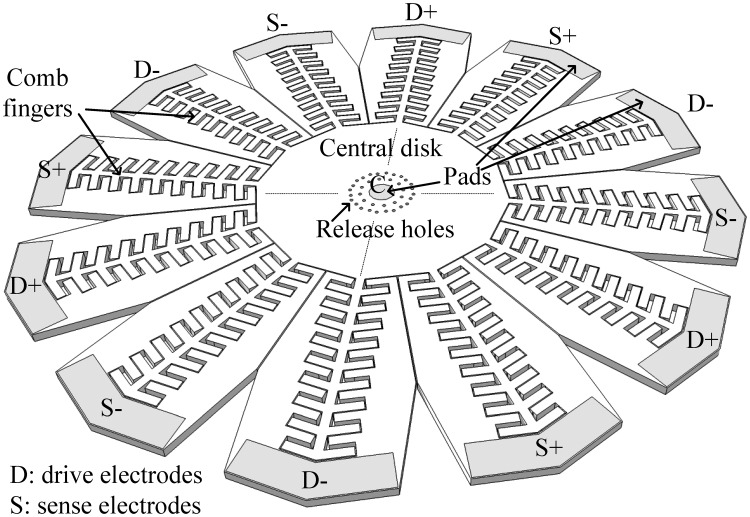
Schematic of the proposed gyro design (for clarity, not all the release holes are shown).

The proposed design is shown in Figure 6, as detailed in [12]. The structure is composed of a central dodecagon structure with a 730 μm face to face distance and a 25 μm thickness. The reason for choosing a dodecagon structure is that the design is implemented in the SOIMUMPs technology, where the device layer is (100) single crystalline silicon (SCS), which is anisotropic. In a disk with the same central disk dimensions, the first order bulk mode exhibits a 1 MHz split between the drive and sense modes. Therefore, in order to have closer matching of the drive and sense modes, the second order bulk mode of a dodecagon (12 sides) structure is favored, providing a drive-sense mode separation of only 270 Hz. The drive and sense modes for the second order mode are spatially separated by 30°. Therefore, a 12-side structure is selected, in order to have 30° between its vertices, so that the second order maxima coincide with the disk vertices. Comb fingers of 25 μm width were added to the vertices, which are the points that exhibit the largest vibration amplitudes. The dimensions of the fingers were optimized, as discussed in the following section, to ensure a sufficiently large stiffness. As was previously discussed, this allows the forces applied to the fingers to be conveyed efficiently to the disk vertices because of the sufficiently high comb spring constant, and also ensures that the combs vibrate constructively with the disk in the wanted senses/drive modes—not in any spurious modes. As shown in Figure 6, the resonating structure is surrounded by electrodes that electrostatically drive the structure (marked as “D”) and sense its output signal differentially (marked as “S”). The central pad (marked as “C”) is used to connect the necessary DC bias voltage to the disk structure. This precludes the need for suspended upper traces as was used in [1–3] or for a lower metallization—both would result in a more complex fabrication process. In order to release the structure, release holes with 10 μm diameter and spaced out by 25 μm are added. The release holes are distributed around the structure in a symmetric manner, in order to mitigate any frequency splits that may arise due to these holes. In addition, the release holes are distanced from the central pad, in order to ensure that a complete release of the disk can be performed, without etching the sacrificial layer under the central pad.

**Figure 7.**
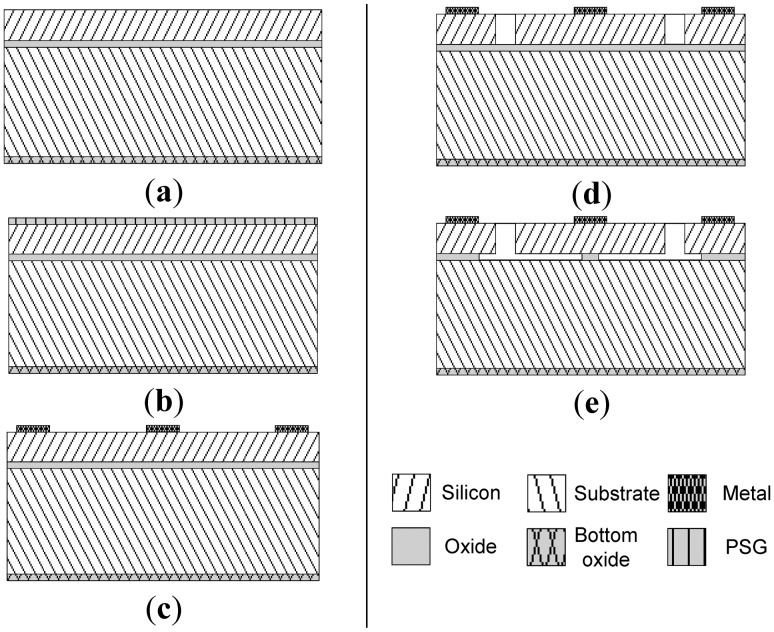
Simplified processing steps of the SOIMUMPs technology [15].

The gyroscopes were fabricated in the MEMSCAP SOIMUMPs technology. The fabrication steps of the SOIMUMPs technology are briefly outlined in Figure 7 [15]. The gyro is fabricated on a double-side polished SOI substrate with a 25 μm (100) SCS structural layer (Figure 7a). The top layer is then doped by depositing phosphosilicate glass (PSG) (Figure 7b), which is then removed by wet etching. Afterwards, a pad metal layer is deposited and patterned by a liftoff process to form the metal pads for the electrical connections as shown in Figure 7c. Then, the device layer is lithographically patterned and etched using deep reactive ion etching (DRIE) to form the proof mass and electrodes (Figure 7d). Finally, the release was performed in-house by timed hydrofluoric acid (HF) wet etching (Figure 7e). An etch rate of 1.6 μm/min is stated in [16] for SOIMUMPs, using 48% aqueous HF and a Triton X-100 surfactant. To validate this etch rate, HF etch tests were performed in-house on similar SOI wafers and an etch rate of ∼1 μm/min using 49% aqueous HF was measured. Careful timing is essential so as to avoid etching below the central pad, which may lead to the structural failure of the disk while wire bonding to it later on, but the timed etch must be long enough to release the structure: both the disk and combs. It would be advantageous to make the central support as small as possible in order to reduce the energy losses due to the coupling of vibrations to the substrate through the central support and thus increase the quality factor of the device. However, the size of the central support in the design is limited to the central pad size, as mentioned above, which limits the quality factor of the device.

## 4. Simulation Results

**Figure 8.**
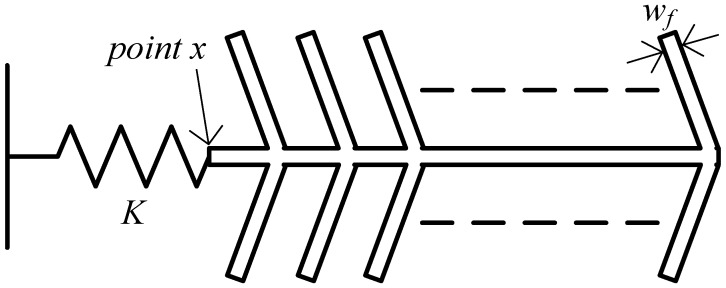
Simplified model of a single comb, where the disk is replaced with a spring of constant K.

The COMSOL Multiphysics finite element simulation package is used for all simulations. As was previously mentioned, for the device to function properly, the comb fingers should be stiff enough so that the electrostatic force applied to them is coupled to the disk faces, and to ensure that they vibrate constructively with the disk and not in any spurious mode. In order to optimize the width of the comb fingers (*w_f_*), the simplified model for one comb drive shown in Figure 8 is used. In this model, the disk is replaced by a spring, with spring constant *K* acting as the primary spring constant in the model in Figure 4, which was obtained to be ∼10^6^ N/m through stationary simulation, by applying a force to the disk faces and measuring the resulting displacement. Stationary simulation was then performed on the model in Figure 8, and the displacement of the comb end which is connected to the spring (point *x*) was measured and compared to that of the tips of the fingers. The finger width was then tuned such that these displacements were almost the same, and scaled according to the number of fingers on which the force was applied. Table 1 shows the stationary displacement of point *x* normalized with respect to the applied force per unit area using Equation (12) for different finger widths:

(12) $${\text{Displacement}}_{\text{normalized}}=\frac{{\text{Displacement}}_{x}}{\text{force per unit area applied}}$$

**Figure 9.**
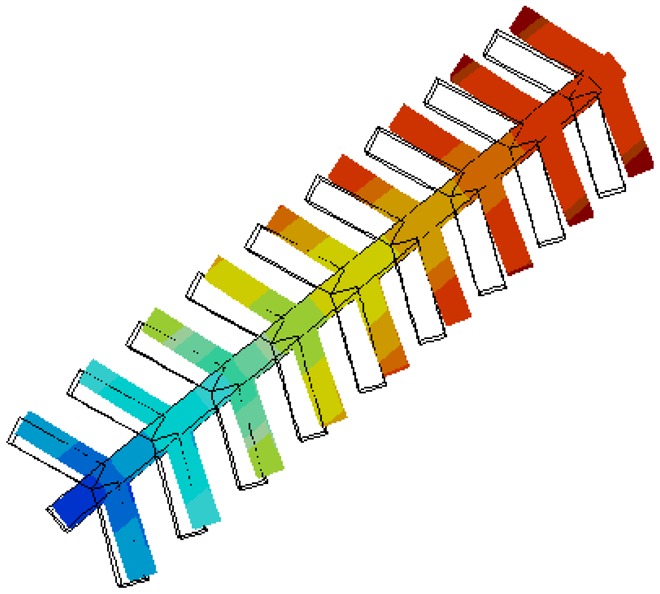
Displacement resulting from applying face forces on the comb fingers.

Based on this analysis, a width of 25 μm was selected for use in the design. This sizing ensures that the forces applied to the fingers are coupled to the end of the comb, and thus can actuate the disk effectively, as shown in Figure 9. Also, it acts as a good compromise between force coupling and device area.

**Figure 10.**
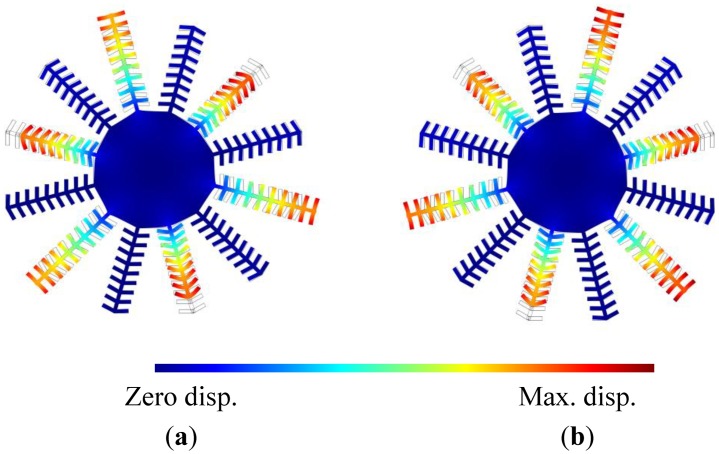
Mode shape of the (**a**) drive mode at 1.499271 MHz, and (**b**) sense mode at 1.499543 MHz, obtained by finite element simulations.

Eigen frequency simulations were performed for the entire disk and comb structure. The resonance frequencies of the drive and sense modes were found to be 1.499271 MHz and 1.499543 MHz, respectively. The mode shapes of the drive and sense modes are shown in Figure 10. It is clear that the combs vibrate constructively with the disk. This was confirmed by performing a frequency analysis for the structures and making sure that the displacement of the disk vertices scale with the number of fingers subject to the face forces.

## 5. Measurement Results

**Figure 11.**
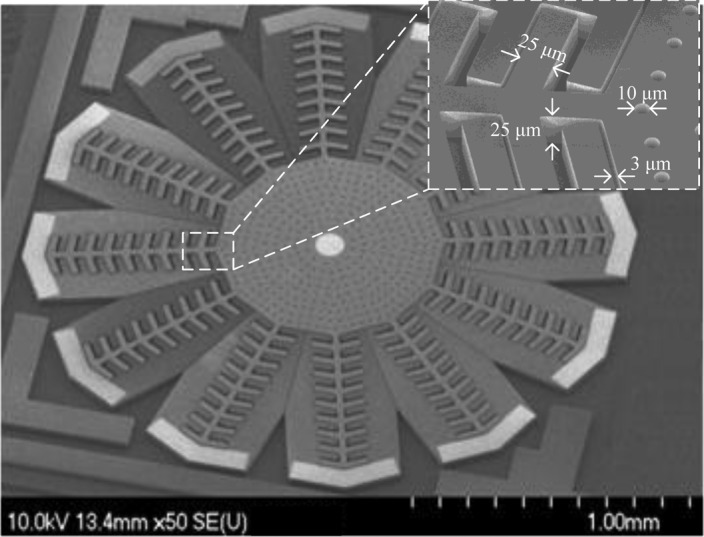
SEM micrograph of a fabricated device with zoomed view of the comb structure.

Devices were fabricated in the SOIMUMPs technology from MEMSCAP. An SEM micrograph of a fabricated device is shown in Figure 11.

**Figure 12.**
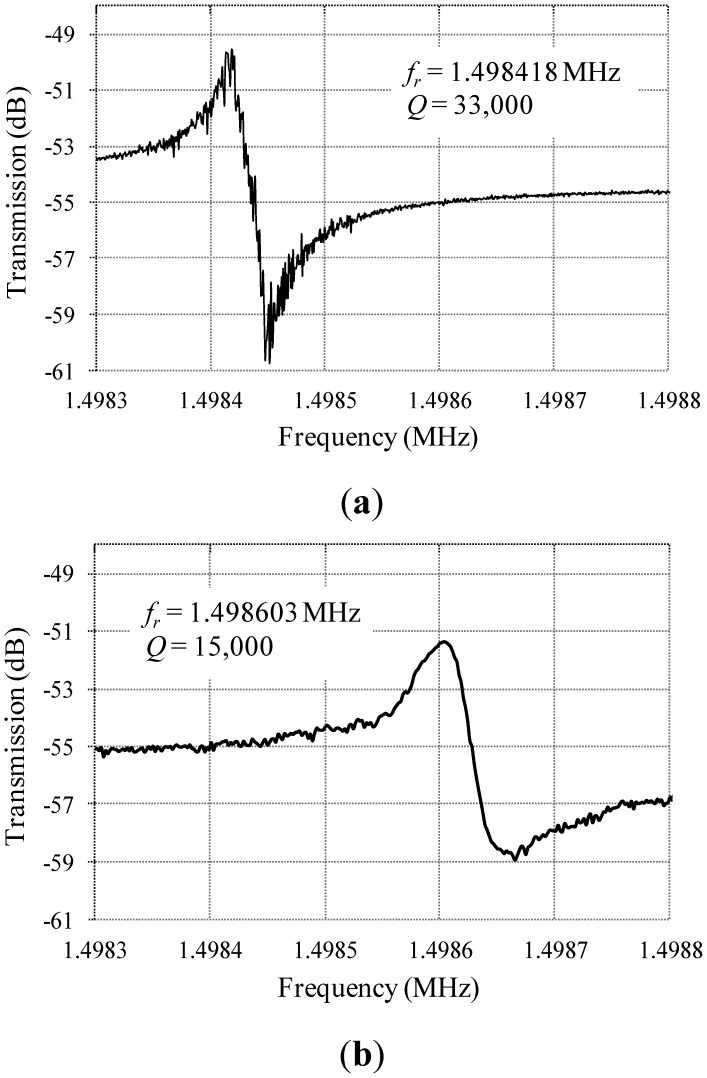
Measured resonance characteristics of the (**a**) drive mode, and (**b**) sense mode (10 mTorr vacuum).

The resonance characteristics of the drive and sense modes were measured under a vacuum level ∼10 mTorr. The prototypes were found to resonate in the drive mode with a center frequency of 1.498418 MHz, and a quality factor of 33,000, and in the sense mode with a center frequency of 1.498603 MHz, and a quality factor of 15,000, as shown in Figure 12. These resonance frequencies are consistent with the simulated results. The difference between the quality factors of the drive and sense modes may be due to the anisotropic elastic properties of the (100) single crystalline silicon structural layer provided by the fabrication technology, as detailed in [17].

**Figure 13.**
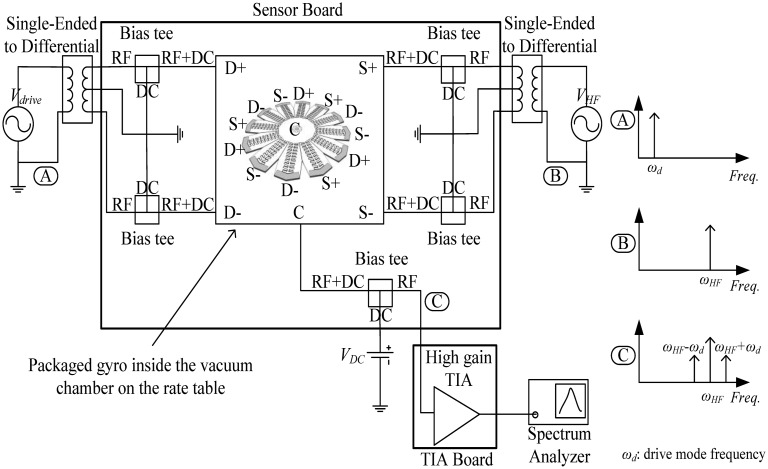
Schematic of the test setup showing the signal frequency spectrum at important nodes.

**Figure 14.**
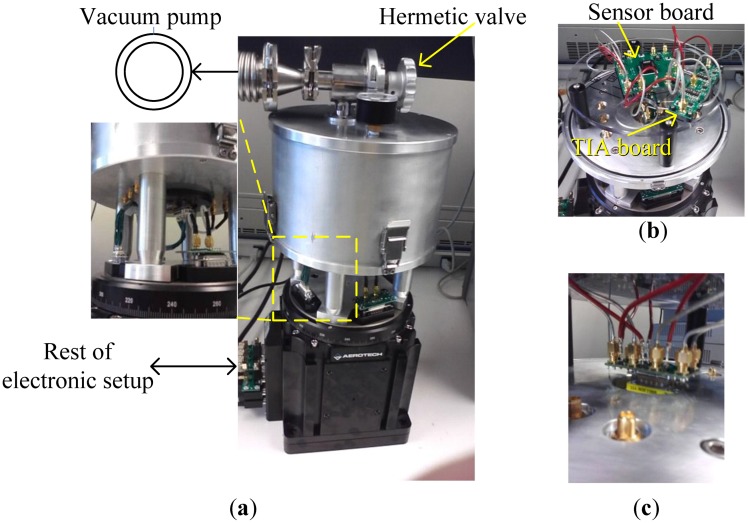
(**a**) Photograph of the test setup, (**b**) circuits and connections from inside the vacuum chamber, and (**c**) vacuum sealed electrical feed-throughs.

**Figure 15.**
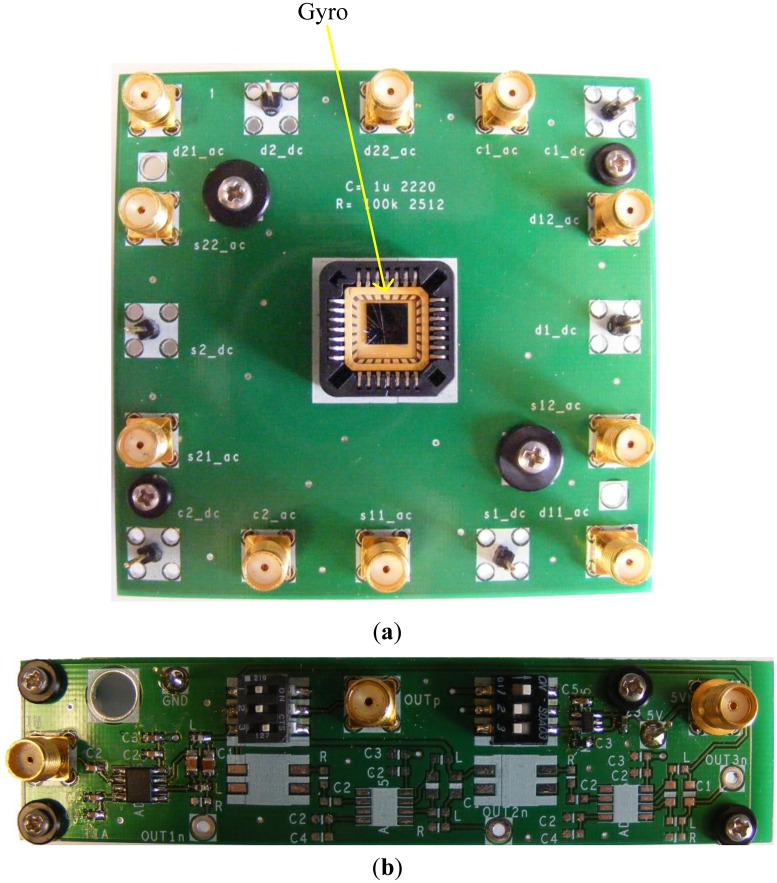
Photographs of (**a**) PCB with packaged gyro, and (**b**) PCB with TIA.

**Figure 16.**
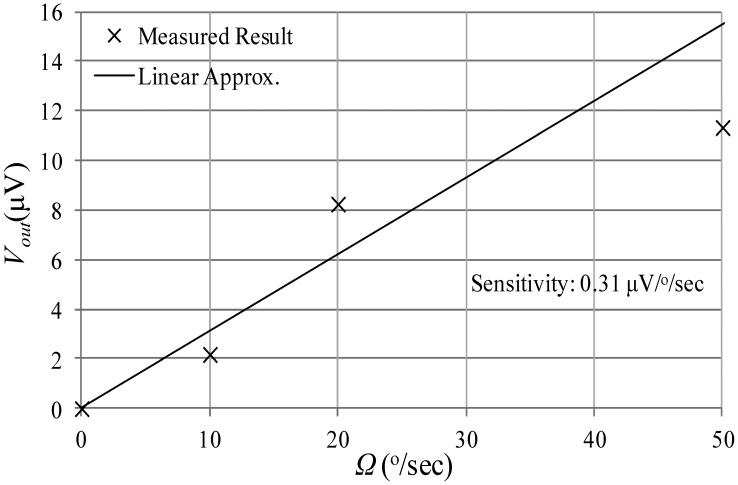
Measured angular rate response, and a linear approximation.

The angular rate response of a prototype gyro was measured at a ∼10 mTorr vacuum level using discrete electronics, a rate table, and a vacuum chamber, fixed on top of the rate table. A schematic of the test setup is shown in Figure 13. The vacuum chamber was designed and custom machined in-house. A hermetic valve was added to it, in order to maintain the vacuum level while rotation after the valve is closed and the pump is disconnected, as shown in Figure 14. Bias tees are used to apply the DC voltages required by the drive and sense electrodes. They are built using discrete components on the sensor board. Furthermore, a high gain trans-impedance amplifier board was built using the AD8015 transimpedance amplifier (TIA) chip from Analog Devices, in order to amplify the gyro's output signal current. Figure 15 shows a photograph of the printed circuit boards (PCBs) that include the gyro and TIA, where the electrodes of the gyro and the input and output of the TIA are each connected to an SMA connector to be interfaced within the test setup. The high frequency signal (*V_HF_*) is used to modulate the output signal of the gyroscope to a higher frequency, in order to separate it from any feed-through from the drive signal utilizing a mixing configuration similar to that in [5,6]. Measurements were limited below 50 °/s angular rate input due to the mechanical limitations of the current test setup. The rate sensitivity was measured to be 0.31 μV/°/sec, corresponding to a capacitance sensitivity of 0.43 aF/°/s/electrode. The resulting measured angular rate response is shown in Figure 16. These measurement results are limited by the parasitics and noise floor of the discrete test setup. Improved performance could be achieved by using an application specific integrated circuit (ASIC) and wire bonding it to the device within the same package to reduce noise and parasitics (e.g., as demonstrated in [18] for a capacitive accelerometer).

## 6. Discussion

A comparison between the design proposed here, and state-of-the-art bulk mode gyros is shown in Table 2. The proposed design, with added combs, achieves two orders of magnitude higher sensitivity than the design presented in [8], of similar shape but without combs, and fabricated in the same technology.

The structure exhibits lower sensitivity than the gyros presented in [1–3], due to their comparatively much smaller transducer gaps. However, based on the 3 μm transducer gap results presented in this work, this gyro structure has the potential of yielding higher sensitivities than the devices in [1–3], when implemented in similar ≾200 nm gap technologies. In order to present a meaningful comparison between the different devices, an appropriate figure of merit (FOM; is introduced:

(13) $$\text{FOM}=\left[\text{Sensitivity per Electrode}\right]\times {\left[g\times 10^{6}\right]}^{2}$$

This FOM is used to normalize the sensitivity with respect to the gap size, as the electrostatic driving force and capacitance variation are proportional to *1*/*g^2^* as shown in Equations (3) and (4) for the variable gap electrode configuration utilized here, and equal electrostatic drive forces for the devices in comparison. The FOM, as it considers the transducer gap differences between the different presented gyros, shows the potential of the improved design to yield a higher sensitivity than conventional designs when fabricated in a given technology.

## 7. Conclusions

This work introduces a novel method for enhancing the sensitivity of bulk mode gyroscopes. It is based on adding parallel plate comb drives to the points of maximum vibration amplitude, and tuning the stiffness of the combs for optimal driving and sensing of the disk's vibrational modes. This proposed method increases the drive strength and results in significant improvement in the sensitivity of the device. This gyro architecture is well-suited to technologies with ∼100 nm-scale transducer gap sizes in order to achieve very high performance devices. Alternatively, the suggested method can also enable the fabrication of high performance bulk-mode gyroscopes that perform adequately in more standard commercial MEMS technologies (e.g., with μm-scale gaps). In this work, the proposed structure was implemented in SOIMUMPs as a proof of concept carried out in a standard large gap size commercial bulk micro-fabrication technology. Prototypes were measured to operate at frequencies of ∼1.5 MHz, with quality factors of up to ∼33,000, at a 10 mTorr vacuum level. Measurements using discrete electronics show a rate sensitivity of 0.31 μV/°/s, corresponding to a capacitance sensitivity of 0.43 aF/°/s/electrode, two orders of magnitude higher than a similar design without combs that were also fabricated in the same technology [8]. This improved performance validates the enhancement potential of the proposed combined disk-comb gyroscope structure over traditional implementations of bulk-mode gyros.

## Figures and Tables

**Table 1.** Normalized displacement at point x for different finger width, w_f_.

**Number of Finger Pairs**	**Normalized Displacement (m^3^/N)×10^14^**			

	**Finger Width,*w****_f_***(μm)**			

	***5***	**10**	***25***	***35***
1	0.35	0.37	0.41	0.44
2	0.68	0.71	0.81	0.88
3	1.01	1.06	1.22	1.32
4	1.34	1.41	1.62	1.76
5	1.67	1.76	2.03	2.2
6	1.99	2.1	2.4	2.64
7	2.32	2.45	2.83	3.08
8	2.65	2.8	3.25	3.52
9	2.98	3.15	3.7	3.96

**Table 2.** Comparison between the comb/bulk mode gyroscope proposed in this work and state-of-the-art.

	**References**					
	
	**[1]**	**[2]**	**[3]**	**[8]**	**This Work**	
Diameter (mm)	0.8	1.2	1.12	0.73	2.13	
Gap (*g*) (μm)	0.25	0.18	0.2	3	3	
Thickness (μm)	50	35	60	25	25	
	
					Drive Sim.	Sense Sim.
	
*f* (MHz)	5.88	2.917	3.12	8.14	1.499271 Meas.	1.499543 Meas.
					1.498418	1.498603
*Q* (,000)	12	66	1	10	33 (meas.)	15 (meas.)
Meas. Rate Sensitivity (μV/°/s)	190	320	15	-	0.31	
Sensitivity per Electrode (aF/°/s)	-	4.63	-	0.002	0.43	
**FOM (μm^2^×aF/°/s)**	**-**	**0.15**	**-**	**0.018**	**3.87**	
